# Thirteenth Annual Report of the Managers of the Buffalo State Asylum for the Insane

**Published:** 1884-04

**Authors:** 


**Thirteenth Annual Report of the Managers of the Buffalo State Asylum for the Insane, Transmitted to the Legislature Jan. 15, 1884.**

The following is from the report of the Superintendent, Dr. Judson B. Andrews:

There was a history of syphilis in fourteen cases, while it was stated as the direct cause of insanity in three cases.

Two hundred and fifty-nine cases were admitted to the asylum for the first time, and only six for the second time. In two hundred and thirty-six cases the present was the first attack; in twenty-two, it was the second; in five, the third; in one, the fourth, and in one the number could not be ascertained.

As regards the physical condition of those admitted, four had hemiplegia, one had chorea, and twenty-two were sick and very feeble. Three had cut throat, one a wound in the breast, one was deaf and dumb, one blind in both eyes, and three in one eye. Two were deaf and one was an idiot. Two were pregnant. Thirteen were debilitated by intemperate habits, and five by vicious habits and indulgences.

Of the two hundred and sixty-five admissions, four were received from other asylums, the rest came directly from their homes or from the counties chargeable with their support.

Fourteen were cases of epilepsy, and eighteen of paresis. Forty had been insane for two or more years, and in twenty-five cases the duration of insanity, though chronic, was unascertained. This makes the prospect for recovery unfavorable in thirty-seven per cent. of the admissions.

We republish in the JOURNAL the following tables, taken from the report of the superintendent, as particularly interesting to physicians:

### TABLE NO. 1.

*General statistics of the asylum.*

| | Men. | Women. | Total. |
|---|---|---|---|
| Patients in the asylum Sept. 30, 1882 | 146 | 128 | 274 |
| Admitted during the year | 139 | 126 | 265 |
| Total in the asylum | 285 | 254 | 539 |
| Discharged recovered | 35 | 30 | 65 |
| much improved | 9 | 7 | 16 |
| improved | 23 | 14 | 37 |
| unimproved | 20 | 21 | 41 |
| died | 33 | 12 | 45 |
| not insane | 6 | .. | 6 |
| Total | 126 | 84 | 210 |
| Remaining in the asylum Sept. 30, 1883 | 159 | 170 | 329 |

| | |
|---|---|
| Maximum number within the year | 339 |
| Minimum | 269 |
| Daily average | 301¾ |
| Ratio of recoveries to number of admissions | 24.5 |
| Ratio of recoveries to average population | 21.6 |
| Ratio of recoveries to number discharged | 31 |

### TABLE NO. 2.

*Received on first or subsequent admissions.*

| No. of admission. | Men. | Women. | Total. |
|---|---|---|---|
| First | 137 | 122 | 259 |
| Second | 2 | 4 | 6 |
| Total cases | 139 | 126 | 265 |

### Table. No. 3.

*Showing the number of attacks in two hundred and sixty-five cases.*

| | Men. | Women. | Total. |
|---|---|---|---|
| First attack | 125 | 111 | 236 |
| Second attack | 10 | 12 | 22 |
| Third attack | 3 | 2 | 5 |
| Fourth attack | 1 | .... | 1 |
| Several attacks | .... | 1 | 1 |
| | 139 | 126 | 265 |

### Table No. 4.

*Annual admissions, discharges and deaths.*

| | Admitted. | | | Disch'ged. Recover'd. | | | Much improved. | | | Im-proved. | | | Unim-proved. | | | Died. | | | Not insane. | | |
|---|---|---|---|---|---|---|---|---|---|---|---|---|---|---|---|---|---|---|---|---|---|
| | Men. | Women. | Total. | Men. | Women. | Total. | Men. | Women. | Total. | Men. | Women. | Total. | Men. | Women. | Total. | Men. | Women. | Total. | Men. | Women. | Total. |
| 1881 .. | 122 | 97 | 219 | 13 | 6 | 19 | 4 | 3 | 7 | 2 | 2 | 4 | 6 | 4 | 10 | 14 | 8 | 22 | 1 | .. | 1 |
| 1882 ... | 157 | 116 | 273 | 30 | 25 | 55 | 9 | 6 | 15 | 11 | 2 | 13 | 28 | 20 | 48 | 10 | 6 | 16 | 5 | 3 | 8 |
| 1883 ... | 139 | 126 | 265 | 35 | 30 | 65 | 9 | 7 | 16 | 23 | 14 | 37 | 20 | 21 | 41 | 33 | 12 | 45 | 6 | .. | 6 |
| Total . | 418 | 339 | 757 | 78 | 61 | 139 | 22 | 16 | 38 | 36 | 18 | 54 | 54 | 45 | 99 | 57 | 26 | 83 | 12 | 3 | 15 |

### Table No. 5.

*General statistics of the asylum from its opening.*

| | |
|---|---|
| Total number of admissions | 757 |
| Total number discharged recovered | 139 |
| Total number discharged much improved | 38 |
| Total number improved | 54 |
| Total number unimproved | 99 |
| Total number died | 83 |
| Total number not insane | 15 |
| Total number | 428 |
| Remaining September 30, 1883 | 329 |

### Table No. 6.

*Form of insanity in two hundred and sixty-five cases admitted.*

| | Men. | Women. | Total. |
|---|---|---|---|
| Acute mania | 37 | 47 | 84 |
| Melancholia | 33 | 30 | 63 |
| Dementia | 32 | 20 | 52 |
| Chronic mania | 4 | 8 | 12 |
| Sub-acute mania | 4 | 3 | 7 |
| Paroxysmal mania | 2 | 4 | 6 |
| Epilepsy with mania | 1 | 3 | 4 |
| Epilepsy with dementia | 6 | 4 | 10 |
| Paresis | 14 | 4 | 18 |
| Not insane | 6 | 3 | 9 |
| Total | 139 | 126 | 265 |

### TABLE No. 7.

*Assigned causes in two hundred and sixty-five cases admitted.*

| | Men. | Women. | Total. |
|---|---|---|---|
| Ill-health from grief and anxiety, overwork or loss of sleep | 25 | 23 | 48 |
| General ill-health | 13 | 26 | 39 |
| Ill-health from want | 2 | 1 | 3 |
| Ill-health from fright | 1 | 2 | 3 |
| Puerperal | .. | 8 | 8 |
| Change of life | .. | 3 | 3 |
| Menstrual irregularities | .. | 5 | 5 |
| Prolonged lactation | .. | 2 | 2 |
| Abortion | .. | 2 | 2 |
| Uterine disease | .. | 1 | 1 |
| Fevers—malarial, typhoid and scarlet | 2 | 2 | 4 |
| Intemperance | 9 | 4 | 13 |
| Masturbation | 2 | .. | 2 |
| Syphilis | 2 | 1 | 3 |
| Vicious habits and indulgences | 5 | .. | 5 |
| Opium habit | .. | 2 | 2 |
| Bright's disease | .. | 1 | 1 |
| Meningitis | 3 | .. | 3 |
| Sunstroke | 2 | .. | 2 |
| Rheumatism | 1 | .. | 1 |
| Phthisis | 1 | .. | 1 |
| Injury to head | 6 | 1 | 7 |
| Apoplexy | 5 | 1 | 6 |
| Locomotor ataxia | 1 | .. | 1 |
| Senility | 2 | .. | 2 |
| Epilepsy | 9 | 6 | 15 |
| Paresis | 14 | 4 | 18 |
| Not insane | 6 | 3 | 9 |
| Unascertained | 28 | 28 | 56 |
| Total | 139 | 612 | 265 |

### TABLE No. 10.

*Of hereditary transmission in two hundred and sixty-five cases admitted.*

| | Men. | Women. | Total. |
|---|---|---|---|
| Maternal branch | 6 | 16 | 22 |
| Paternal branch | 6 | 6 | 12 |
| Paternal and maternal branches | 5 | .. | 5 |
| Insane relations | 13 | 11 | 24 |
| Total | 30 | 33 | 63 |

### TABLE No. 12.

*Ages of two hundred and sixty-five cases admitted.*

| | Men. | Women. | Total. |
|---|---|---|---|
| From 10 to 15 years | 2 | 1 | 3 |
| 15 to 20 years | 14 | 9 | 23 |
| 20 to 25 years | 16 | 13 | 29 |
| 25 to 30 years | 13 | 13 | 26 |
| 30 to 35 years | 18 | 21 | 39 |
| 35 to 40 years | 15 | 17 | 32 |
| 40 to 50 years | 27 | 26 | 53 |
| 50 to 60 years | 20 | 18 | 38 |
| 60 to 70 years | 7 | 8 | 15 |
| 70 to 80 years | 5 | .. | 5 |
| 80 to 90 years | 2 | .. | 2 |
| Total | 139 | 126 | 265 |

### Table No. 16.

*Ages of sixty-five cases recovered.*

| | Men. | Women. | Total. |
|---|---|---|---|
| From 15 to 20 | 5 | 2 | 7 |
| 20 to 25 | 5 | 3 | 8 |
| 25 to 30 | 5 | 6 | 11 |
| 30 to 35 | 6 | 7 | 13 |
| 35 to 40 | 1 | 6 | 7 |
| 40 to 50 | 9 | 4 | 13 |
| 50 to 60 | 3 | 2 | 5 |
| 60 to 70 | 1 | .. | 1 |
| Total | 35 | 30 | 65 |

### Table No. 17.

*Form of insanity in sixty-five cases recovered.*

| | Men. | Women. | Total. |
|---|---|---|---|
| Acute mania | 17 | 19 | 36 |
| Melancholia | 11 | 6 | 17 |
| Sub-acute mania | 2 | 1 | 3 |
| Chronic mania | 1 | 1 | 2 |
| Paroxysmal mania | 1 | .. | 1 |
| Dementia | 3 | 2 | 5 |
| Epilepsy with mania | .. | 1 | 1 |
| Total | 35 | 30 | 65 |

### Table No. 18.

*Causes of death in forty-five cases.*

| | Men. | Women. | Total. |
|---|---|---|---|
| Paresis | 16 | 1 | 13 |
| Heart disease | 2 | 1 | 7 |
| Exhaustion | 2 | 1 | 3 |
| Chronic diarrhœa | 2 | 1 | 3 |
| Apoplexy | 3 | 1 | 4 |
| Cerebral hemorrhage | 3 | 1 | 4 |
| Erysipelas | .. | 1 | 1 |
| Epilepsy | .. | 1 | 1 |
| Chronic bronchitis | 1 | .. | 1 |
| Bright's disease | .. | 1 | 1 |
| Strangulation | 1 | .. | 1 |
| Suspension | 1 | .. | 1 |
| Pneumonia | 1 | 1 | 2 |
| Cancer of uterus | .. | 1 | 1 |
| Hemorrhage of the lungs from gangrene | 1 | .. | 1 |
| Pyæmia | .. | 1 | 1 |
| Total | 33 | 12 | 45 |

### Table No. 19.

*Ages of the forty-five who died.*

| | Men. | Women. | Total. |
|---|---|---|---|
| From 15 to 20 years | .. | 1 | 1 |
| 20 to 25 years | .. | 1 | 1 |
| 25 to 30 years | .. | 2 | 2 |
| 30 to 40 years | 3 | 1 | 4 |
| 40 to 50 years | 12 | 3 | 15 |

| | Men. | Women. | Total |
|---|---|---|---|
| 50 to 60 years | 5 | 2 | 7 |
| 60 to 70 years | 7 | 2 | 9 |
| 70 to 80 years | 4 | .. | 4 |
| 80 to 90 years | 2 | .. | 2 |
| Total | 33 | 12 | 45 |

TABLE No. 20.

*Form of insanity in forty-five cases of death.*

| | Men. | Women. | Total. |
|---|---|---|---|
| Acute mania | 3 | 1 | 4 |
| Melancholia | 5 | 4 | 9 |
| Chronic mania | 1 | 2 | 3 |
| Dementia | 7 | 3 | 10 |
| Epilepsy with dementia | 1 | 1 | 2 |
| Paresis | 16 | 1 | 17 |
| Total | 33 | 12 | 45 |

TABLE No. 21.

*Conditions associated with eighteen cases of paresis admitted during the year.*

| | Men. | Women. | Total. |
|---|---|---|---|
| Syphilis | 4 | .. | 4 |
| Intemperance | 1 | .. | 1 |
| Syphilis and intemperance | 1 | .. | 1 |
| Uncomplicated or unknown | 8 | 4 | 12 |
| Total | 14 | 4 | 18 |

The report of the superintendent is one of unusual interest and includes remarks on the following topics: Asylum building, water supply, service department, how organized, etc. The first subject is the kitchen, with a dietary arranged for the week, then the laundry, engine houses, shops, etc., the farm officers of asylum, including night watcher and other attendants, with an ingenious blank for keeping the nightly record, school, medical care and supervision, visits to and reception of patients, labor accomplished and amusement furnished. We learn also from another source that Drs. Granger and Crego, the assistants to the superintendent, have instituted a training school for nurses more particularly devoted to instruction in the care of the insane.

